# Coxsackievirus infection induces a non-canonical autophagy independent of the ULK and PI3K complexes

**DOI:** 10.1038/s41598-020-76227-7

**Published:** 2020-11-04

**Authors:** Yasir Mohamud, Junyan Shi, Hui Tang, Pinhao Xiang, Yuan Chao Xue, Huitao Liu, Chen Seng Ng, Honglin Luo

**Affiliations:** 1 Centre for Heart Lung Innovation, St. Paul’s Hospital, 1081 Burrard St., Vancouver, BC V6Z 1Y6 Canada; 2 Department of Pathology and Laboratory Medicine, University of British Columbia, Vancouver, BC Canada; 3 Department of Pharmacy, Shandong Provincial Hospital Affiliated to Shandong First Medical University, Jinan, People’s Republic of China

**Keywords:** Biochemistry, Cell biology, Microbiology

## Abstract

Coxsackievirus B3 (CVB3) is a single-stranded positive RNA virus that usurps cellular machinery, including the evolutionarily anti-viral autophagy pathway, for productive infections. Despite the emergence of double-membraned autophagosome-like vesicles during CVB3 infection, very little is known about the mechanism of autophagy initiation. In this study, we investigated the role of established autophagy factors in the initiation of CVB3-induced autophagy. Using siRNA-mediated gene-silencing and CRISPR-Cas9-based gene-editing in culture cells, we discovered that CVB3 bypasses the ULK1/2 and PI3K complexes to trigger autophagy. Moreover, we found that CVB3-induced LC3 lipidation occurred independent of WIPI2 and the transmembrane protein ATG9 but required components of the late-stage ubiquitin-like ATG conjugation system including ATG5 and ATG16L1. Remarkably, we showed the canonical autophagy factor ULK1 was cleaved through the catalytic activity of the viral proteinase 3C. Mutagenesis experiments identified the cleavage site of ULK1 after Q524, which separates its N-terminal kinase domain from C-terminal substrate binding domain. Finally, we uncovered PI4KIIIβ (a PI4P kinase), but not PI3P or PI5P kinases as requisites for CVB3-induced LC3 lipidation. Taken together, our studies reveal that CVB3 initiates a non-canonical form of autophagy that bypasses ULK1/2 and PI3K signaling pathways to ultimately converge on PI4KIIIβ- and ATG5–ATG12–ATG16L1 machinery.

## Introduction

Enteroviruses (EVs) are a group of single-stranded positive RNA viruses of the *Picornaviridae* family accounting for many diseases, such as various neurological disorders, including poliomyelitis, aseptic meningitis, encephalitis, and non-polio flaccid paralysis, particularly in infants and children worldwide. Since the effective vaccination of poliovirus (PV), non-polio EVs, such as coxsackievirus B3 (CVB3), EV-A71, and EV-D68, have become the most common EVs associated with these diseases, for which effective therapeutic strategies are lacking^1^.

Autophagy is usually regarded as an anti-viral mechanism that selectively degrades viral particles and/or its components (termed virophagy)^2^. However, accumulating evidence indicates that EVs subvert cellular autophagy to facilitate productive infection^3^. Almost all major EVs, including CVB3^4,5^, PV^6^, EV-A71^7^, and EV-D68^8^, have been shown to facilitate the production of autophagosomes, as evidenced by LC3 lipidation/puncta and the appearance of double-membraned vesicles. It has recently been established that CVB3 and EV-D68 trigger the formation of autophagosomes but disrupt the degradative capacity of autophagy by targeting SNARE proteins critical for autophagosome-lysosome fusion for degradation^8–10^. This viral strategy benefits virus by escaping viral RNA/protein from autophagy degradation and facilitating viral replication/release, consequently causing impaired clearance of protein aggregates that are harmful to the host^3^. Despite increasing knowledge about the interplay between EVs and the host autophagy pathway, the exact mechanism by which EVs initiate autophagosome biogenesis remains largely unclear.

Autophagy is canonically recognized as being regulated by nutrient status. Under basal conditions, components of the uncoordinated (UNC)-51-like kinase (ULK) complex, including ULK1/2 and autophagy related 13 (ATG13) undergo inhibitory phosphorylation via the nutrient sensing kinase mechanistic target of rapamycin complex 1 (mTORC1). Upon starvation, ULK1/2 dissociate from mTORC1 to initiate activating phosphorylation of ATG13, RB1 inducible coiled-coil 1 (RB1CC1), and ATG101. Downstream of the ULK complex is the class III phosphoinositide 3 kinase (PI3K) complex composed of beclin 1 (BECN1), ATG14, phosphatidylinositol 3-kinase (PIK3) catalytic subunit 3 (PIK3C3), PIK3 regulatory subunit 4 (PIK3R4), and activating molecule in beclin-1-regulated autophagy protein 1 (AMBRA1). This secondary complex phosphorylates PI lipids of the phagophore to recruit phosphoinositide-3-phosphate (PI3P)-interacting proteins such as WD repeat domain phosphoinositide-interacting 2 (WIPI2), ATG2, and transmembrane protein ATG9. A key event in the biogenesis of autophagosomes is the recruitment of ubiquitin-like conjugating enzymes that participate in the covalent attachment of microtubule associated protein 1 light chain 3 (MAP1LC3/LC3/ATG8) to phosphatidylethanolamine (PE) lipids on the nascent membrane. Newly translated proLC3 is quickly processed by the cysteine protease ATG4 to generate cytosolic LC3-I. Upon autophagy induction, LC3-I is shuttled from the E1-like enzyme ATG7 and E2-like enzyme ATG3 before being anchored to PE (to become LC3-II) through the E3-like activity of ATG5–ATG12–ATG16L complex^11,12^.

In the current study, we aimed to systematically evaluate the role of canonical autophagy factors in CVB3-induced autophagy. Using a combination of LC3 lipidation and puncta formation as markers of autophagy induction, we showed that CVB3-induced autophagy was dependent on the ATG ubiquitin-like conjugation system but bypassed the requirement of canonical ULK and PI3K complexes. Interestingly, we further demonstrated that CVB3 targets several autophagy proteins involved in the canonical autophagy pathway for degradation. Finally, we investigated the possible mechanism of this non-canonical autophagy pathway.

## Results

### CVB3-induced LC3 puncta and lipidation require ATG5 and ATG16L1

**Figure 1:**
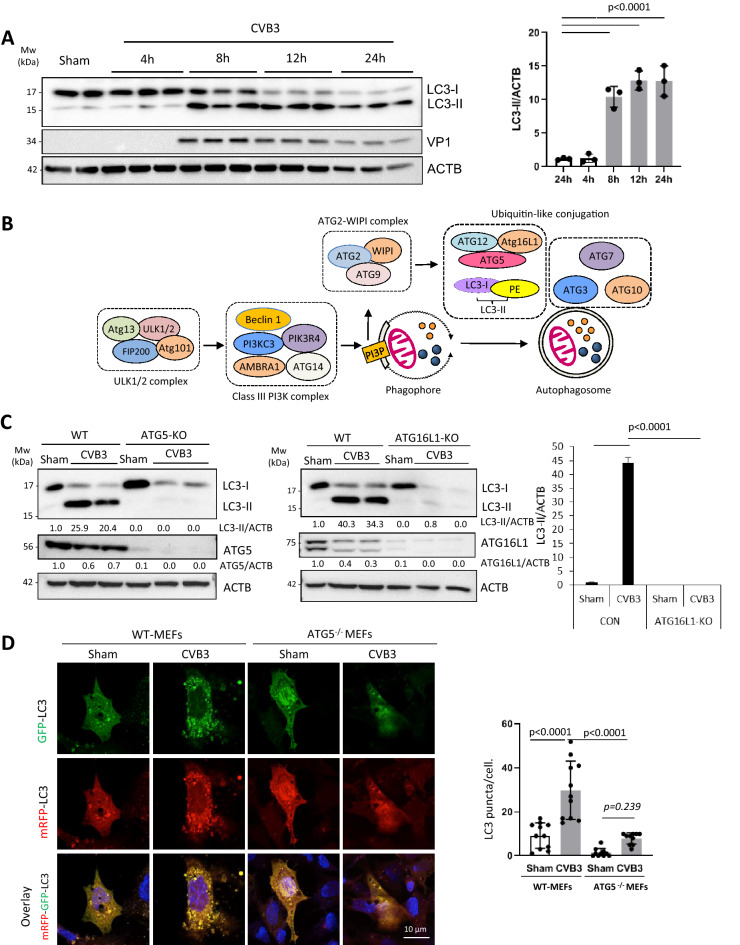
CVB3-induced LC3 puncta and lipidation are dependent on ATG5 and ATG16L1. (**A**) HEK293A were either sham-infected with PBS or infected with CVB3 for indicated time-points. Western blotting was performed to detect LC3 lipidation and ACTB expression (loading control). Levels of LC3-II were quantified by densitometric analysis, normalized to ACTB and presented as fold changes in the right panel (mean ± SD, n = 3, analyzed by one-way ANOVA with Tukey’s post-test). (**B**) Schematic diagram of canonical autophagy pathway induced by amino acid deprivation. (**C**) ATG5- or ATG16L1-knockout (KO) HEK293A cells generated via CRISPR-Cas9 editing, or wildtype (WT)-HEK293A cells were either sham- or CVB3-infected for 16 h. Cell lysates were harvested and probed for LC3, ATG5, and ATG16L1 by Western blotting. Densitometric analysis was carried out as above (the first lane is arbitrarily set a value of 1) and the results are presented under each blot. (**D**) WT- or Atg5^−/−^ MEFs transfected with mRFP–GFP–LC3 construct were sham- or CVB3-infected for 8 h. GFP and RFP signal was captured by confocal microscopy. Mean LC3 puncta per cell was quantified (n ≥ 10 cells) and analyzed by one-way ANOVA with Tukey post-test (right).

To measure the effect of CVB3 infection on autophagy initiation, human embryonic kidney (HEK293A) cells were selected due to their low levels of background autophagy under basal conditions^13,14^. HEK293A cells were infected with CVB3 for different time-points. Protein expression of autophagosome-associated LC3-II, a marker of autophagosomes, was analyzed by western blotting. Figure 1A demonstrated that, as compared to sham-infected cells, there was a significant increase in LC3-II protein levels upon CVB3 infection, starting at ~ 8 h and persisting for up to 24 h post-infection. Virus-induced accumulation of LC3-II was confirmed in neuronal cells, the murine motorneuron-like NSC-34 cells and human neuroblastoma SH-SY5Y cells (***Supplemental Figure 1). To determine the molecular mechanism by which CVB3 triggers autophagy, we first examined the role of canonical autophagy factors in this process. The established upstream autophagy pathways induced by amino acid deprivation are illustrated in Fig. 1B. A previous study made the surprising observation that Vaccinia virus can induce LC3 lipidation independent of ATG5 and ATG7^15^. Thus, we initially investigated whether ubiquitin-like conjugation systems are required for CVB3-induced LC3 lipidation. Utilizing the CRISPR-Cas9 system, we knocked out (KO) ATG5 or ATG16L1 in HEK293A cells. As shown in Fig. 1C, compared to wild-type (WT) cells, CVB3 infection failed to induce LC3-II formation in both ATG5-KO and ATG16L1-KO cells, suggesting an ATG5- and ATG16L1-dependent process. This finding was confirmed in ATG5^−/−^ mouse embryonic fibroblasts (MEFs) transiently transfected with mRFP–GFP–LC3 plasmid that has been widely used to monitor autophagic flux^16^. Figure 1D showed that, in WT-MEFs, CVB3 infection led to a marked increase of cells with LC3 puncta. However, cells positive for LC3 puncta were significantly reduced when ATG5 was deleted. Together, these results suggest that unlike Vaccinia virus, CVB3-induced LC3 lipidation and puncta require the ubiquitin-like conjugation enzymes of the autophagy pathway.

### CVB3-induced LC3 lipidation occurs independent of FIP200 and ATG13

**Figure 2:**
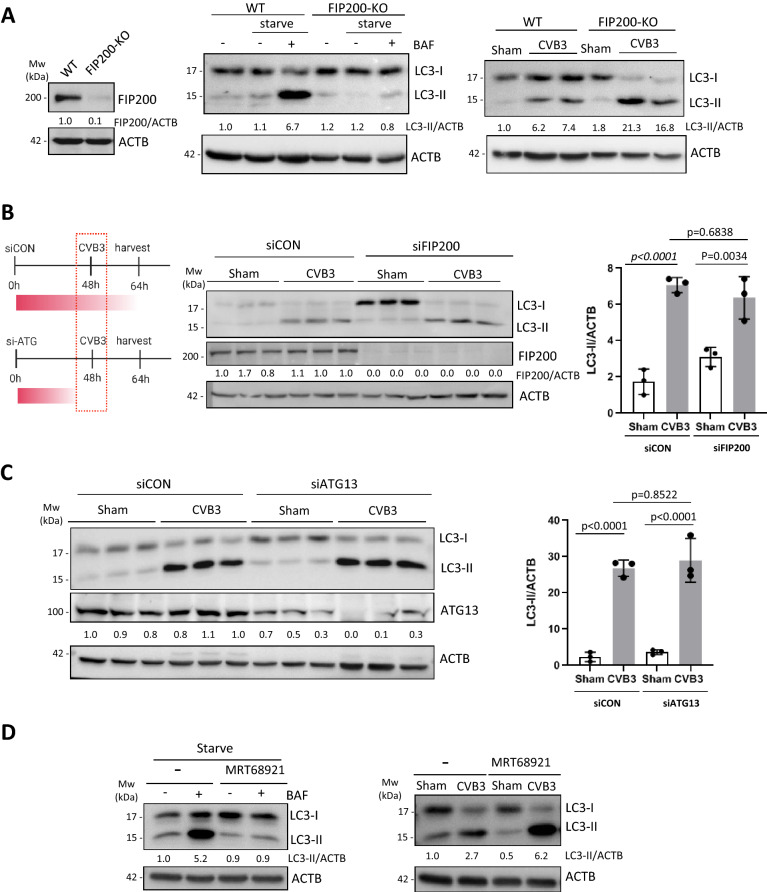
CVB3-induced LC3 lipidation occurs independent of FIP200 and ATG13. (**A**) FIP200-KO HEK293A cells were established through CRISPR-Cas9 gene editing. Knockout efficiency was validated by western blotting (left panel). WT-HEK293A or FIP200-KO cells were cultured in either normal medium, HBSS starvation medium, or starvation medium supplemented with 200 nM bafilomycin (BAF) for 2 h (middle panel). WT or FIP200-KO cells were sham- or CVB3-infected for 16 h (right panel). Western blotting was performed for analysis of LC3 lipidation. (**B**,**C**) Schematic of siRNA-based gene silencing and CVB3 infection schedule (left), HEK293A cells were transiently transfected with scrambled siRNA control (siCON) or siRNAs against FIP200 (**B**) or ATG13 (**C**) for 48 h. Cells were then subjected to sham or CVB3 infection for an additional 16 h. Western blotting was conducted to determine knockdown efficiency and LC3 lipidation. Densitometry was measured as above and the results are presented either underneath the blots or in the right panel (mean ± SD, n = 3, analyzed by one-way ANOVA with Tukey’s post-test). (**D**) HEK293A cells were treated with a selective ULK1/2 kinase inhibitor (MRT68921, 5 µM) under starvation for 2 h in the presence or absence of 200 nM BAF (left panel). HEK293A cells were sham- or CVB3-infected for 16 h with or without 5 µM MRT68921 (right panel). Western blotting was performed to examine LC3 levels and the quantified results are shown underneath the blots.

Next we sought to more closely address whether CVB3-induced LC3 lipidation utilizes the upstream canonical autophagy initiation machinery. As illustrated in Fig. 1B, during starvation, autophagy induction is regulated by the ULK complex that consists of ULK1/2 kinases, ATG13, RB1CC1/FIP200, and ATG101^12^. RB1CC1/FIP200 is an essential component of the ULK complex and its genetic ablation was previously demonstrated to abrogate the canonical autophagy pathway^17^. FIP200-KO HEK293A cells were generated through CRISPR-Cas9 gene editing (Fig. 2A, left). To recapitulate previous findings and validate our FIP200-KO cells, we performed starvation treatment in the absence or presence of bafilomycin A1 (BAF), a vacuolar-ATPase inhibitor that blocks the autophagosome-lysosome fusion process required for degradative autophagy. Autophagy flux can be measured by comparing the levels of LC3-II in the absence or presence of BAF. As expected, starvation-induced autophagy flux was dramatically impaired in FIP200-KO compared to WT cells, supporting an important role for FIP200 in starvation-induced autophagy (Fig. 2A, middle). It was previously reported that modulation of vesicle acidification, including treatment with BAF1, significantly impairs enteroviral replication^8,18^. To eliminate this confounding variable, CVB3 infected cells were not treated with BAF-A1. We next tested whether loss of FIP200 would have an effect on CVB3-induced LC3 lipidation. Compared to control cells, FIP200-KO HEK293A cells that are infected with CVB3 demonstrated comparable accumulation of LC3-II (Fig. 2A, right). Similar buildup of LC3-II was observed in CVB3-infected cells in which FIP200 was transiently silenced using siRNA as compared to cells treated with a scrambled siRNA (Fig. 2B). To corroborate these observations, we also examined whether gene-silencing of ATG13, another essential component of the ULK complex^19^, would impact CVB3-induced LC3 lipidation. Indeed, knockdown of ATG13 using siRNA resulted in a comparable accumulation of LC3-II in CVB3-infected cells as that observed in control siRNA-treated cells (Fig. 2C). Given that ULK1/2 are required for starvation-induced autophagy, we also tested whether ULK1/2 kinase activity plays a role in CVB3-induced LC3 lipidation using the chemical compound MRT68921, a potent inhibitor of both ULK1/2^20^. Whereas treatment of HEK293A cells with MRT68921 completely abrogated starvation-induced autophagy, similar treatment in CVB3-infected cells did not reduce LC3-II accumulation (Fig. 2D). Collectively, these data suggest that the ULK complex is dispensable for CVB3-induced LC3 lipidation, in agreement with a previous finding with PV^21^.

### CVB3-induced LC3 lipidation is independent of BECN and PIK3C3

**Figure 3:**
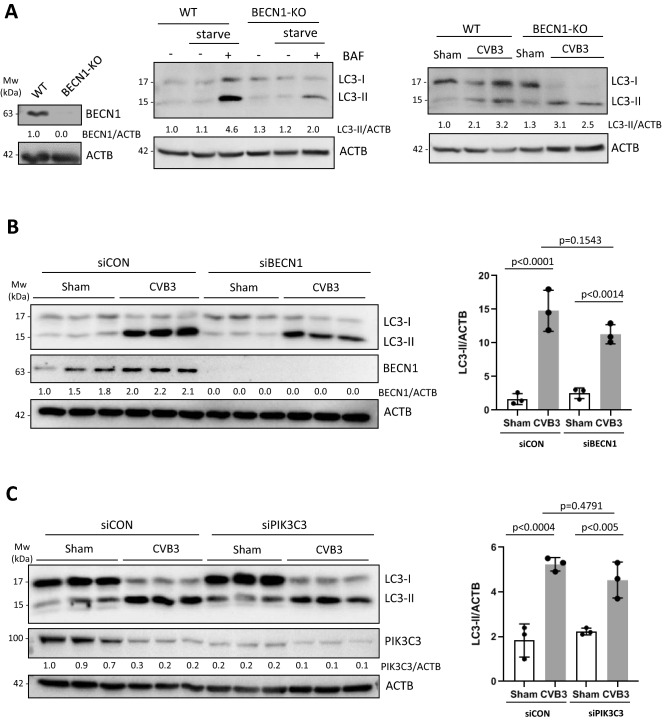
CVB3-induced LC3 lipidation is independent of BECN1 and PIK3C3. (**A**) BECN1-KO HEK293A cells were established through CRISPR-cas9 editing. Knockout efficiency was verified by western blotting (left panel). WT or BECN1-KO cells were cultured in either normal medium, HBSS starvation medium, or starvation medium supplemented with 200 nM BAF for 2 h, followed by western blot analysis of LC3 (middle panel). WT or BECN1-KO cells were sham- or CVB3-infected for 16 h, followed by western blot assessment of LC3 (right panel). (**B**,**C**) BECN1 (**B**) and PIK3C3 (**C**) were transiently silenced in HEK293A cells via siRNA treatment for 48 h. Cells were then subjected to sham or CVB3 infection for 16 h. Cells were harvested and subjected to western blot analysis of LC3, BECN1, and PIK3C3. Densitometric results are presented either underneath the blots or in the right panel (mean ± SD, n = 3, analyzed by one-way ANOVA with Tukey’s post-test).

Downstream of the ULK complex is the PI3P-generating PI3K complex that consists of BECN1, ATG14, PIK3C3, PIK3R4, and AMBRA1 (Fig. 1B)^22^. To determine the possible involvement of the PI3K complex in CVB3-induced LC3 lipidation, BECN1-KO HEK293A cells were generated using CRISPR-Cas 9 approach. Upon verification of the KO efficiency through western blot analysis (Fig. 3A, left), the cells were subjected to starvation treatment in the presence or absence of BAF. Similar to the observation in FIP200-KO cells, starvation-induced autophagy was impaired in BECN1-KO cells (Fig. 3A, middle). However, CVB3-induced LC3-II accumulation was comparable between control and BECN1-KO cells, suggesting that the process is independent of BECN1 (Fig. 3A, right). LC3-II accumulation was also observed in CVB3-infected cells subjected to transient silencing of BECN1 (Fig. 3B) or PIK3C3, the active catalytic subunit of the PI3K complex (Fig. 3C). Taken together, these data suggest that CVB3 infection promotes LC3-II production independent of BECN1 and PIK3C3.

### CVB3-induced LC3 lipidation is independent of ATG9 and WIPI2

**Figure 4:**
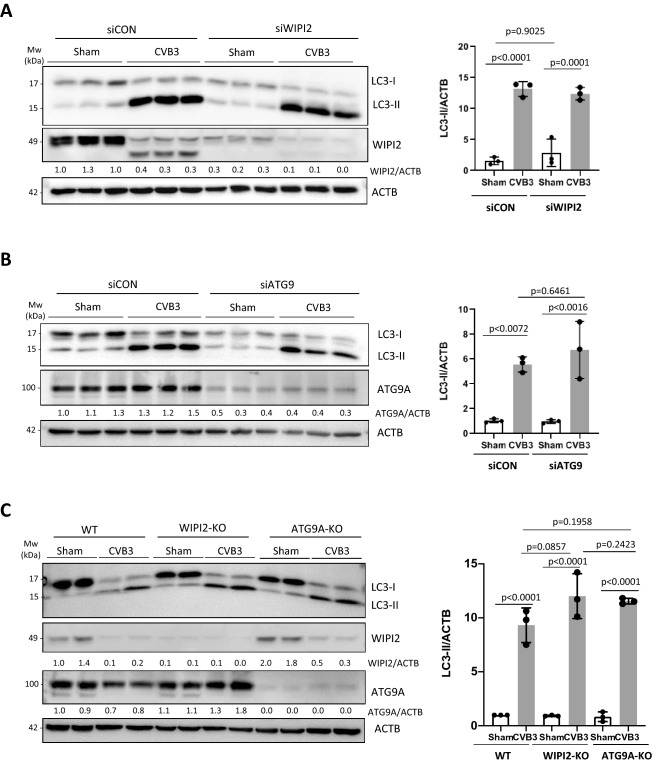
CVB3-induced LC3 lipidation is independent of ATG9 and WIPI2. (**A**,**B**) WIPI2 (**A**) and ATG9A (**B**) were transiently silenced in HEK293 cells through siRNA treatment for 48 h. Cells were then sham- or CVB3-infected for 16 h. Western blotting was conducted to verify the knockdown efficiency and to examine LC3 levels. Densitometric results are presented either underneath the blots or in the right panel (mean ± SD, n = 3, analyzed by one-way ANOVA with Tukey’s post-test). (**C**) WIPI2-KO and ATG9A-KO HEK293A cells, generated through CRISPR-Cas9 gene editing, were sham- or CVB3-infected as above, followed by western blot analysis of LC3, WIPI2, and ATG9A. Densitometry was measured and presented as above.

With the evidence that CVB3-induced autophagy is independent of the ULK1/2 and PI3K complexes, we next set out to address whether CVB3 can bypass upstream autophagy initiators to directly influence autophagosome biogenesis. During canonical autophagy, the activity of the lipid kinase PI3K complex results in the enrichment of autophagic membranes with PI3P. Phosphorylated lipids then serve as recruitment hubs for downstream proteins harboring PI3P interacting domains such as WIPI2 and DFCP1, that may further recruit LC3-lipidation complexes and membranes via ATG9 (Fig. 1B)^23,24^. WIPI2 and ATG9A were transiently silenced in HEK293A cells using siRNAs, followed by sham or CVB3 infection. Similar to the observations above, LC3 lipidation was induced upon CVB3 infection independent of WIPI2 and ATG9 (Fig. 4A,B). Moreover, WIPI2-KO and ATG9A-KO cells generated via CRISPR-Cas9 engineering showed no significant impairment in CVB3-induced LC3 lipidation (Fig. 4C). Collectively, these data indicate that CVB3 initiates autophagy independent of ATG9 and WIPI2.

### Several autophagy proteins are targeted during CVB3 infection

**Figure 5:**
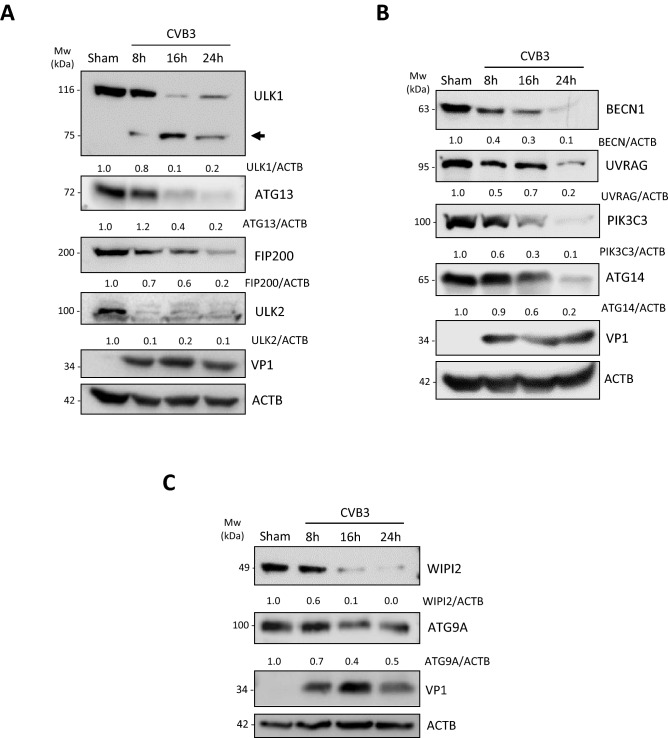
CVB3 targets several autophagy proteins in HEK293A cells. (**A**–**C**) HEK293A cells were sham- or CVB3-infected for 8 h, 16 h, or 24 h as indicated. Cell lysates were harvested and probed for ULK1, ATG13, FIP200, and ULK2, components of the ULK1/2 complex (**A**), BECN1, UVRAG, PIK3C3, and ATG14, components of the PI3K complex (**B**), and WIPI2 and ATG9A, components of the WIPI complex (**C**). Protein levels were quantified and presented underneath each western blot as above.

To investigate the direct effects of CVB3 infection on critical components of the canonical autophagy pathway, we proceeded to closely measure the levels of autophagy proteins during a 24 h time-course of CVB3 infection in HEK293A cells. We first examined components of the ULK1/2 complex and observed a significant reduction in protein levels of ULK1, ULK2, RB1CC1/FIP200, and ATG13. Of note, the protein loss of ULK1 was accompanied by the emergence of lower molecular weight fragments (~ 75 kDa) at ~ 8 h post-infection (Fig. 5A). Similarly, protein levels of major components of the PI3K and WIPI complexes, including BECN1, UVRAG, PIK3C3, ATG14, WIPI2, and ATG9A, were also decreased following CVB3 infection (Fig. 5B,C). The decrease in autophagy proteins following CVB3 infection was most pronounced when cells were infected in the absence of transient transfection as transient transfection was previously reported to impair and/or delay viral replication^25^.

**Figure 6:**
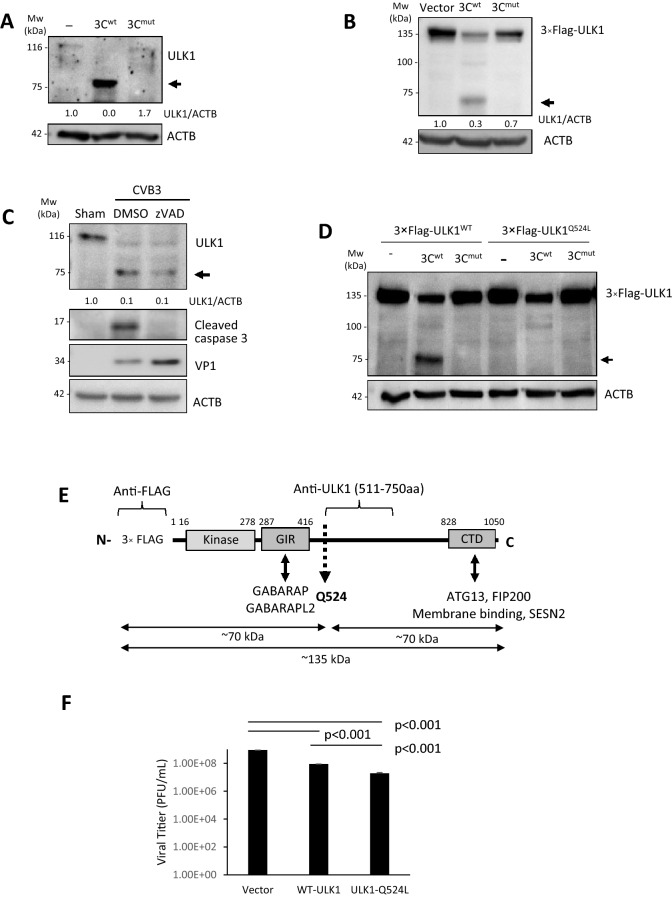
Viral proteinase 3C cleaves ULK1 after glutamine 524. (**A**) In vitro cleavage assay was performed by incubating lysates from HeLa cells with vehicle (−), purified wildtype 3C (3C^wt^), or catalytically inactive 3C (C147A) mutant (3C^mut^) proteins, followed by western blot analysis of ULK1 using an antibody that recognizes an internal region (amino acids 511–750) of ULK1. Arrow denotes the cleavage fragment. (**B**) HeLa cells were transfected with 3 × Flag-ULK1 together with either empty vector, myc-3C^wt^, or myc-3C^mut^ as indicated. After 24 h, cell lysates were collected and analyzed by western blotting with antibodies against FLAG. (**C**) HeLa cells were infected with CVB3 for 7 h in the presence or absence of a pan-caspase inhibitor z-VAD-FMK (zVAD, 50 µM) or DMSO (vehicle). Western blotting was performed with antibodies against ULK1. Activation of caspase-3 was examined using an anti-cleaved caspase-3 antibody. Densitometry was measured as above. (**D**) HeLa cells were co-transfected with 3 × FLAG-ULK1^WT^ or 3 × FLAG-ULK1^Q524L^ (Glutamine 524 mutated to Leucine), together with empty vector, myc-3C^wt^, or myc-3C^mut^. Western blotting was performed with an anti-FLAG antibody. (**E**) Schematic illustration of the structural domains, the identified cleavage site, the antibody recognition regions, and the resulting cleavage products of ULK1. *GIR,* GABARAP interacting region; *CTD,* C-terminal domain. (**F**) HEK293 cells were transfected with either vector control, WT-ULK1 or non-cleavable ULK1 (ULK1-Q524L) for 24 h followed by CVB3 infection for an additional 8 h. Supernatant was collected for viral titer measurement (mean ± SD, n = 3, analyzed by unpaired Student t-test).

The significant reduction in ULK1 protein levels accompanied by the detection of lower molecular weight fragments prompted us to explore the potential role of virus-encoded proteinases. In vitro cleavage assay was performed using cell lysates incubated with either vehicle control, purified WT viral proteinase 3C (3C^wt^) or catalytically inactive 3C (C147A) mutant (3C^mut^). We found that 3C^wt^, but not 3C^mut^, was able to significantly reduce the full-length proform and recapitulate cleavage fragments observed in CVB3-infected cells (Fig. 6A). Similarly, cells co-transfected with 3 × Flag-ULK1 (~ 135 kDa) and myc-tagged 3C^wt^ but not myc-3C^mut^ displayed the 75 kDa-cleavage fragments (Fig. 6B). To exclude the possible role of host caspases that are activated during the late stage of CVB3 infection in the cleavage of ULK1, we utilized the pan-caspase inhibitor zVAD-fmk. We found that caspase inhibition failed to attenuate CVB3-induced cleavage of ULK1 (Fig. 6C). Site-directed mutagenesis was utilized to further identify the site of viral proteinase 3C cleavage of ULK1. Figure 6D showed that ULK1^Q524L^ mutant was resistant to CVB3-induced cleavage, suggesting that the cleavage takes place at this position although it cannot be excluded that additional cleavages may take place. This cleavage led to the separation of the N-terminal kinase domain and the GABARAP interacting region (GIR) from the C-terminal domain (CTD) of substrate binding (Fig. 6E). Finally, we demonstrated that expression of wild-type ULK1 led to impaired viral replication while non-cleavable ULK1^Q524L^ further reduced viral titers, indicating an anti-viral activity for ULK1 (Fig. 6F). Taken together, these data support that viral proteinase 3C is responsible for the cleavage of ULK1 during CVB3 infection.

### PI4KIIIβ is an upstream factor in CVB3-induced autophagy

**Figure 7:**
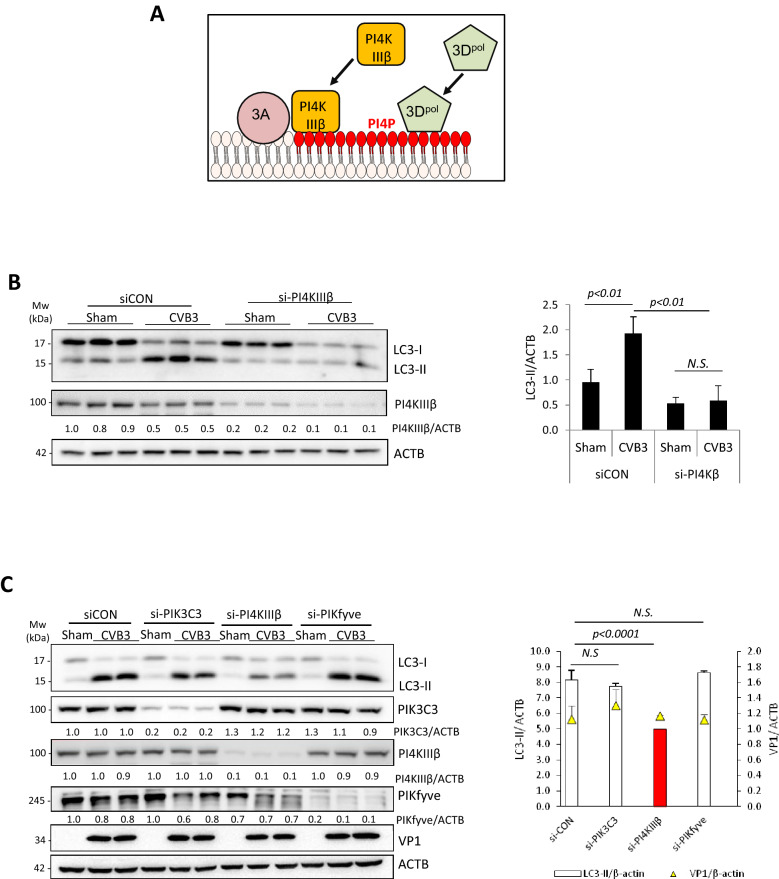
PI4KIIIβ is involved in CVB3-induced LC3 lipidation. (**A**) Schematic diagram of the proposed role of PI4KIIIβ in autophagy and the known function in enterovirus replication. (**B**) PI4KIIIβ was transiently silenced in HEK293A cells using siRNA for 48 h. Cells were then subjected to sham or CVB3 infection for 16 h, followed by western blot analysis of LC3 and PI4KIIIβ. Densitometry was measured as above, and the results are presented underneath and in right panel (mean ± SD, n = 3. Analyzed by one way ANOVA with Tukey’s post-test). (**C**) HEK293A cells, transfected with control or PIK3C3, PI4KIIIβ, or PIKfyve siRNAs for 48 h, were sham- or CVB3-infected. Western blotting was performed for detection of LC3, VP1, PIK3C3, PI4KIIIβ, or PIKfyve, and quantified as above (right panel) (mean ± SD, n = 3. Analyzed by one way ANOVA with Tukey’s post-test). *N.S. *not significant.

Our observations that canonical autophagy factors are dispensable for CVB3-induced autophagy, coupled with the findings that viral proteinase(s) target key autophagy proteins for degradation, suggest the existence of non-canonical, alternate autophagy pathways in initiating CVB3-induced autophagy. In addition to PI3P, both PI4P and PI5P have been identified as alternative phospholipids to induce autophagy^26,27^. During EV infection, membrane-anchored viral protein 3A has been shown to recruit PI4KIIIβ to the viral replication organelles to promote the production of PI4P. PI4P in turn recruits viral polymerase 3D to initiate viral RNA synthesis (Fig. 7A)^28,29^. To test the role of PI4P in CVB3-induced autophagy, we genetically silenced PI4KIIIβ, a major enzyme regulating PI4P synthesis. We discovered that CVB3-induced LC3 lipidation is markedly inhibited in cells depleted of PI4KIIIβ compared to control siRNA-treated cells, suggesting a PI4P-dependent mechanism (Fig. 7B). We next compared the role of PI4KIIIβ to PIK3C3 (a PI3P kinase) and PIKfyve (FYVE finger-containing phosphoinositide kinase, a PI5P kinase) in a single experiment. We found that the viral dose utilized in this study (MOI = 100) had no major influence on viral replication, as evidenced by equal viral protein production among three groups (Fig. 7C). We confirmed that gene-silencing of PI4KIIIβ, but not PIK3C3 or PIKfyve, was responsible for the attenuation of CVB3-induced LC3 lipidation (Fig. 7C).

## Discussion

EVs have been shown to induce autophagosome accumulation as a viral strategy to facilitate productive infection. The initiation of autophagy allows EVs to hijack autophagic membranes as topological surfaces that can be repurposed as replication organelles for viral RNA synthesis^4,6,30^. Additionally, the acidification of autophagosomes/amphisomes has been proposed to act as a maturation chamber for newly synthesized virions^8,18^, which may also commandeer autophagosomes as quasi-envelopes that act as vehicles for non-lytic viral propagation^31–33^. These insights have established a complex relationship between EV and the autophagic process during various stages of the viral life cycle.

The production of double-membraned vesicles, development of LC3 puncta, and accumulation of LC3-II marker following viral infection have been documented for many EVs^4–8^. A recent study has provided evidence that PV-induced autophagy is independent of the ULK complex^21^; however the precise mechanisms by which EVs initiate autophagy remain to be fully elucidated. The current study was designed to use CVB3 as a model system to investigate how EVs trigger autophagosome biogenesis. Lipidation of LC3 by the ubiquitin-like ATG conjugation system plays an essential role in host autophagy by facilitating membrane curvature, recruitment of selective autophagy receptors and/or cargo, closure of autophagosomes, and membrane fusion with endolysosomal compartments^34^. Our present study has uncovered that CVB3-induced LC3 lipidation requires the ATG ubiquitin-like conjugation system, but independent of the canonical starvation-induced upstream signaling pathways such as ULK1/2 signaling and PI3P production. In addition, PI3P-binding effectors, such as WIPI2 that facilitates LC3 lipidation by recruitment of ATG5–ATG12–ATG16L1 complex, were also found to be dispensable for CVB3-induced LC3 lipidation.

Our observations that canonical autophagy factors are not required for CVB3-induced autophagy, coupled with the findings that viral proteinase(s) target key autophagy proteins for cleavage, suggest the existence of non-canonical, alternate autophagy pathways in initiating CVB3-induced autophagy. Several instances of non-canonical autophagy have been previously described, such as ULK1/2-independent autophagy. It was shown that ammonia-induced autophagy is dependent on ATG5, but does not require ULK1/2^35^. Rubicon was identified as a key modulator of ULK-independent, LC3-associated phagocytosis (LAP)^36,37^, a form of ‘non-canonical’ autophagy that occurs in immune cells and utilizes some components of the autophagy machinery (e.g. ATG conjugation machinery) to process extracellular cargo through single membrane endocytic vesicles^38^. Interestingly, we found that the protein levels of Rubicon were undetectable in neural cells and very low in HeLa and HEK293 cells compared to Jurkat cell (not shown), indicating that Rubicon may not play a major role in CVB3-induced autophagy.

Class III PI3K-independent autophagy was also previously reported^27^. It was shown that PIK3C3-dependent PI3P production is dispensable for glucose deprivation-induced autophagy^27^. Further research identified PI5P, synthesized by PIKfyve (FYVE finger-containing phosphoinositide kinase), as an alternative phospholipid to induce autophagy^27^. However, we found that knockdown of PIKfyve was unable to block CVB3-elicited LC3 lipidation (not shown), suggesting that PI5P is not a key autophagy initiating factor upon CVB3 infection.

In addition to PI3P and PI5P, recent studies also suggest a role for PI4P in autophagosome biogenesis. PI4P is a lipid that is predominantly found on the membranes of the trans Golgi network (TGN)^29,39^. It was recently shown that ATG9A recruits PI4KIIIβ, a major enzyme regulating PI4P synthesis, to the autophagosome initiation site for PI4P production and deletion of PI4KIIIβ disrupts the process of autophagy^26^. Interestingly, during CVB3 and PV infection, the replication organelles of viruses were found to be enriched in PI4P, partly through the TGN-anchored viral membrane protein 3A that recruits PI4KIIIβ to the viral replication organelles. Enhanced PI4P production further recruits viral polymerase 3D to initiate viral RNA synthesis^29,40^. Since PI4KIIIβ is needed for effective viral replication, complete knockout will lead to reduced viral growth, which may interfere with the data interpretation of LC3-II accumulation. Therefore, in this study we partially knocked down PI4KIIIβ through siRNA gene-silencing and infected the cells with an MOI of 100. We found that knockdown of PI4KIIIβ significantly reduces the accumulation of LC3-II without affecting viral replication, indicating a role for PI4KIIIβ in CVB3-induced autophagy. Our data also suggest that the PI4P-enriched membrane compartments are likely a source of CVB3-induced autophagosome-like structures. Currently, the autophagy effectors downstream of PI4P remain to be identified. Our finding that knockdown or knockout of WIPI2 does not inhibit CVB3-induced LC3 lipidation excludes WIPI2 as an effector protein of PI4P. Interestingly, ATG16L1 was recently reported to have intrinsic PI4P-lipid binding capacity through its central coiled-coil domain^41^, suggesting that ATG16L1 may directly respond to local production of PI4P at replication organelles.

In conclusion, our study reveals that CVB3 initiates a novel form of autophagy depending on ATG5–ATG12–ATG16L1 complex and PI4KIIIβ-PI4P pathway, but being distinct from the physiological, starvation-induced ‘canonical’ autophagy. The current study adds CVB3 as a novel stimulus in the emerging field of non-canonical autophagy.

## Materials and methods

### Cell culture, viral infection, and chemicals

HEK293A and HeLa were from American Type Culture Collection (ATCC). ATG5^−/−^ and wild-type (WT) mouse embryonic fibroblasts (MEF) were kind gifts from Dr. Noboru Mizushima^42^, and obtained through BIKEN BioResource Research Center (RCB2711 and RCB2710, respectively). These cells were cultured in Dulbecco’s Modified Eagle’s Medium (DMEM) supplemented with 10% fetal bovine serum (FBS) and a penicillin/streptomycin cocktail (100 µg/mL).

For CVB3 infection, cells were either sham-infected with PBS or inoculated with CVB3 (Kandolf strain) at a multiplicity of infection (MOI) of 100 for HEK293A and MEF cells and 10 for HeLa cells. The following chemicals were used for the treatment of cells: general caspase inhibitor Z-VAD-FMK (BD Biosciences, #550377), lysosome inhibitor bafilomycin A1 (Sigma-Aldrich, B1793), ULK1/2 kinase inhibitor MRT68921 HCl (Selleck, S7949). Cells were starved by culturing in Hank’s Balanced Salt Solution (HBSS) medium (Thermofisher Scientific, 14025076) for 2 h.

### Generation of knockout cells using CRISPR-Cas9 system

The following knockout (KO) cell lines were generated using the CRISPR-Cas9 system as previously described^43^: ATG5, ATG16L1, FIP200, BECN1, WIPI2, and ATG9A HEK293A KO cells. The guide RNA (gRNA) sequences are as follows: ATG5: 5′-GAACTTGTTTCACGCTATATC-3′; ATG16L1: 5′-GATTCTCTGCATTAAGCCGAT-3′; FIP200: 5′-CAGGTGCTGGTGGTCAATGG-3′; BECN1: 5′-GCACACGAAGCTCACCTGCA-3′; WIPI2: 5′-GCAGCTACTCCAACACGATTC-3′; ATG9A: 5′-GCCTGTTGGTGCACGTCGCCG-3′. The gRNA was cloned into pSpCas9 (BB)-2A-Puro vector (Addgene #62988) following digestion with BbsI. Positive clones were isolated by single-cell selection following puromycin treatment (2–7 μg/mL). Knockout efficiency was verified by western blot analysis.

### Plasmids and small interfering RNA (siRNA)

The myc-tagged wild-type CVB3-3C (3C^wt^) and C147A mutant CVB3-3C (3C^mut^) constructs were generous gifts from Dr. Carolyn Coyne at the University of Pittsburgh^44^. The mRFP–GFP–LC3 plasmid was a gift from Dr. Tamotsu Yoshimori (Addgene, 21074).

The 3 × Flag-ULK1 plasmid was generated using a multiple cloning site modified CMV10 vector backbone with the corresponding cut site (EcorI/BamHI). Mutant 3 × Flag-ULK1 Q524L was generated using a gBLOCKS fragment (Integrated DNA Technologies) harbouring the point mutation A1571T in the ULK1 coding sequence and cloned using restriction enzymes FseI and AflII. The scrambled siRNA (sc-37007) and the siRNAs targeting FIP200 (sc-38211), ATG13 (sc-97013), BECN1 (sc-29797), PIK3C3 (sc-62802), WIPI2 (sc-72212), ATG9 (sc-72586), and PIKfyve (sc-39142) were purchased from Santa Cruz Biotechnology. The siRNA targeting PI4KIIIβ was obtained from Dharmacon (D-006777-02). For transfection, cells were transiently transfected with plasmid cDNAs or siRNAs using Lipofectamine 2000 (Invitrogen, 11668-019) following the manufacturer’s instructions.

### Western blot analysis

Cells were lysed in buffer (10 mM HEPES pH 7.4, 50 mM NaPyrophosphate, 50 mM NaF, 50 mM NaCl, 5 mM EDTA, 5 mM EGTA, 100 µM Na_3_VO_4_, 0.1% Triton X-100) and Western blotting was conducted using the following primary antibodies: LC3B (Novus Biologicals, NB100-2220), β-actin (ACTB, Sigma-Aldrich, A5316), ATG5 (Santa Cruz, sc-33210), FIP200 (Cell Signaling Technology, D10D11), BECN1 (Santa Cruz, sc-48341), PIK3C3 (Santa Cruz, sc365404), WIPI2 (Cell Signaling Technology, #8567), ATG13 (Cell Signaling Technology, D4P1K), ATG9A (Cell Signaling Technology, D4O9D), ULK1 (Santa Cruz, sc-390904), ULK2 (GeneTex, GTX111476), UVRAG (Santa Cruz, sc-293268), ATG14 (Cell Signaling Technology, #5504), ATG16L1 (Santa Cruz, sc-393274), cleaved-caspase 3 (Cell Signaling Technology, #9661), viral capsid protein 1 (VP1, Dako, M706401-1), FLAG (Sigma, F1804), PI4KIIIβ (Sigma 06578), and PIKfyve (Santa Cruz, sc-100408). Blots were cropped to improve clarity and conciseness. Uncropped blots are provided in Supplemental Figure 2.

### In vitro cleavage assay

In vitro cleavage assay was performed as previously described^45^. Briefly, HeLa cell lysates (30 µg) were incubated with purified WT or catalytically inactive CVB3 proteinase 3C (0.1 µg) in a cleavage assay buffer (20 mM HEPES pH 7.4, 150 mM potassium acetate, and 1 mM DTT) for 16 h at 37 °C. Reaction was terminated with 6 × SDS sample buffer, followed by 95 °C denaturation and subsequent western blot analysis.

### Confocal microscopy

After fixation and PBS washes of the cells, coverslips were mounted using Fluoroshield with 4, 6-diamidino-2-phenylindole (DAPI, Sigma-Aldrich, F6057). Images were captured with the Zeiss LSM 880 Inverted Confocal Microscopy. LC3 puncta per cell was quantified using the Spot Detector plugin in the open source bio-imaging software Icy 1.9.5.0 as previously described^46^. Puncta per cell was averaged from 3 biologically independent experiments and presented in scatter plot.

### TCID50 assay

Supernatant samples were serially diluted and overlaid on 60-well Terasaki plates of HeLa cells. After 48 h incubation, 50% tissue culture infective dose titer (TCID50) was calculated and viral titers were expressed as plaque-forming unit (PFU)/mL with one infectious unit equal to 0.7 TCID50.

### Statistical analysis

Results are presented as mean ± standard deviation (SD). Statistical analysis was performed with unpaired Student’s *t *test or analysis of variance (ANOVA) using GraphPad Prism 5 Software. A P-value < 0.05 was considered to be statistically significant. All results presented are representative of at least 3-averaged independent experiments.

## Supplementary information

Supplementary information.
