# Supplementary material for: Coxsackievirus infection induces a non-canonical autophagy independent of the ULK and PI3K complexes
Source: Sci Rep. 2020 Nov 4;10:19068. doi: 10.1038/s41598-020-76227-7 (PMC7642411; doi:10.1038/s41598-020-76227-7)
Supplement: Supplementary file 1 — Supplementary information. [file 41598_2020_76227_MOESM1_ESM.docx]

**SUPPLEMENTARY INFORMATION**

**Coxsackievirus infection induces a non-canonical autophagy independent of the ULK and PI3K complexes**

Yasir Mohamud^1,2^, Junyan Shi^1,2^, Hui Tang^1,3^, Pinhao Xiang^1,2^, Yuan Chao Xue^1,2^, Huitao Liu^1,2^, Chen Seng Ng^1,2^, Honglin Luo^1,2*^

^1^Centre for Heart Lung Innovation, St. Paul's Hospital, Vancouver, BC, Canada

^2^Department of Pathology and Laboratory Medicine, University of British Columbia, Vancouver, BC, Canada

^3^ Department of Pharmacy, Shandong Provincial Hospital Affiliated to Shandong First Medical University

^*^Correspondence:

Honglin Luo, Centre for Heart Lung Innovation, St. Paul’s Hospital, 1081 Burrard St., Vancouver, BC V6Z 1Y6, Canada. Email: [honglin.luo@hli.ubc.ca](mailto:honglin.luo@hli.ubc.ca)

**
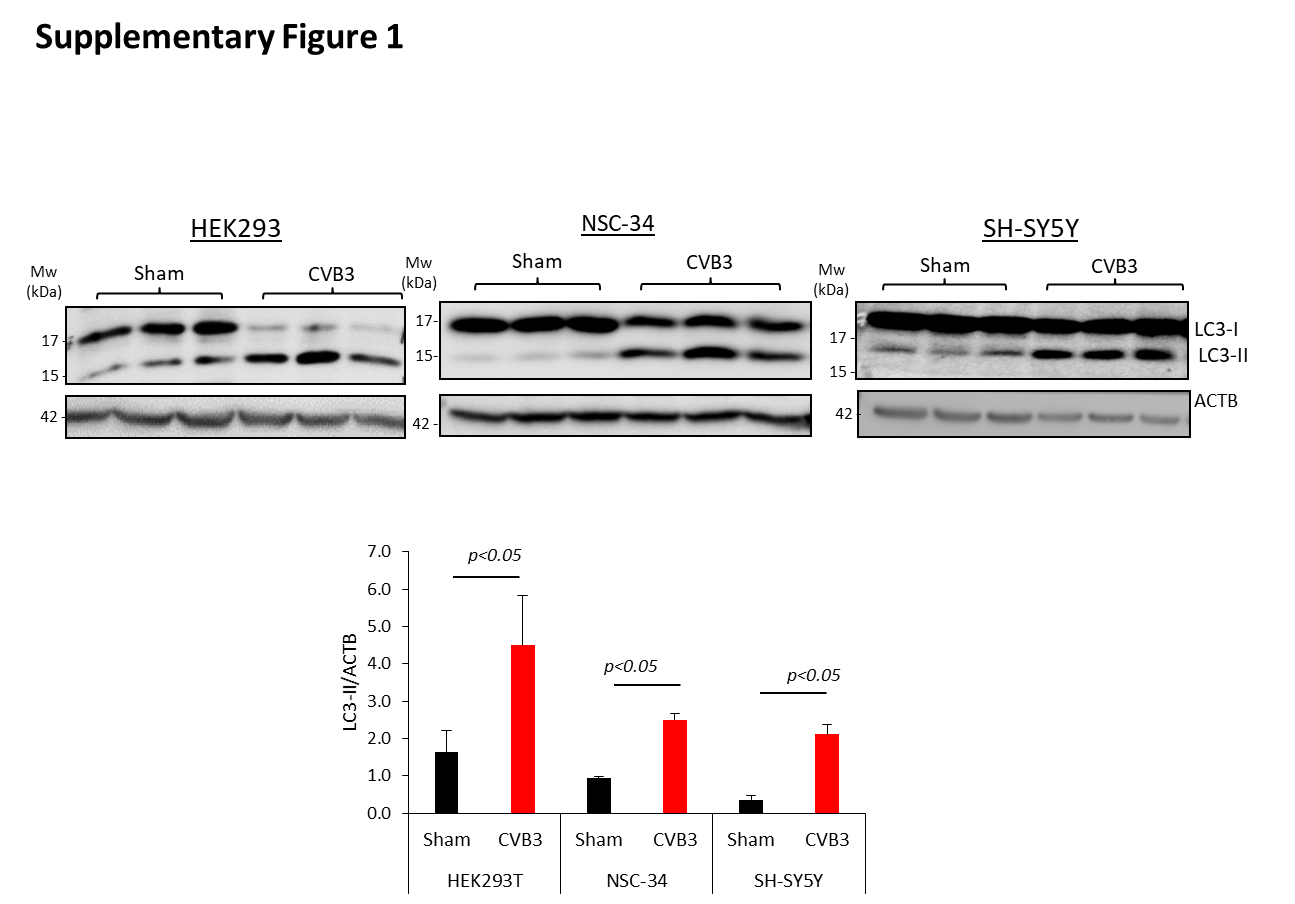
**

**Supplementary Figure 1. CVB3-induced LC3 accumulation in HEK293, NSC-34, and SH-SY5Y cells.**

HEK293A, NSC-34, and SH-SY5Y cells were sham- or CVB3-infected for 16h. Cell lysates were harvested for western blot analysis. Autophagy induction was assayed by LC3-II conversion, normalized to ACTV and quantified in the bottom panel (mean±SD, n=3, analyzed by unpaired Student t-test).
